# HIF-1α and BMAL1 in bone regeneration: crosstalk between hypoxia response and circadian rhythm

**DOI:** 10.1038/s41413-026-00506-8

**Published:** 2026-02-14

**Authors:** Yihang Weng, Jiong Xiong, Qing Zhao, Zhen Tan

**Affiliations:** 1 Department of Implantology, State Key Laboratory of Oral Diseases, National Clinical Research Center for Oral Diseases, West China Hospital of Stomatology, Sichuan University, Chengdu, Sichuan Province China; 2 Department of Orthodontics, State Key Laboratory of Oral Diseases, National Clinical Research Center for Oral Diseases, West China Hospital of Stomatology, Sichuan University, Chengdu, Sichuan Province China

**Keywords:** Bone, Metabolism

## Abstract

Bone regeneration is initiated after a bone injury, such as a bone fracture or tooth extraction. It is a highly complex biological process involving multiple cell types, signaling molecules, and molecular pathways. The hypoxic microenvironment in the early stage of bone regeneration poses challenges to cell status and the final outcome of bone regeneration. During this phase, two key regulators—HIF-1α (the critical mediator of hypoxia response) and BMAL1 (the central component of the circadian rhythm)—orchestrate the activities of bone-regenerating cells, ensuring proper cellular function and orderly progression of bone repair. Existing studies have shown that there is a close crosstalk between HIF-1α and BMAL1, including regulation of gene expression, protein interaction, and regulation of downstream pathways. In this review, we discuss the respective regulatory roles of HIF-1α and BMAL1 in bone regeneration and further summarize their interactions within cells. Additionally, we extend the discussion to their interactions in other bone-related diseases, and summarize the existing research directions and deficiencies, providing new insights for in-depth studies of the hypoxia response and circadian rhythm systems.

## Introduction

Bone is a metabolically active organ, wherein numerous diverse cell types actively participate in intricate bone metabolic processes. This enables the maintenance of structural and functional stability of the bone, as well as its capacity for self-repair and regeneration when injured.^1,2^ Bone regeneration is commonly observed in the wound following bone fracture or tooth extraction.^3,4^ The structure, continuity, and function of the local bone tissue in both conditions are disrupted, necessitating initiation of the repair process, execution of bone remodeling, and completion of bone regeneration. Investigating the mechanisms influencing bone regeneration to further regulate and promote this process represents a current research focus.

The process of bone regeneration is a highly intricate biological process, encompassing various cellular types, signaling molecules, and molecular pathways. It follows a precise temporal and spatial sequence to accomplish the induction and conduction of bone formation, ultimately leading to the complete repair of bone structure and restoration of functionality.^5–7^ Bone regeneration involves a series of metabolic processes and can be described by three overlapping phases: the inflammatory phase, the proliferative phase, and the modeling phase.^3,8^ Among them, the response during the inflammatory phase significantly determines the ultimate outcome of bone healing.^9^ Hypoxia resulting from vascular disruption activates specific transcription factors essential to the repair process, thereby inducing cellular metabolic reprogramming.^10–12^ The intercellular communication between immune cells and bone mesenchymal stem cells (BMSCs) plays a pivotal role in regulating this process.^8,13^ Therefore, the identification of key molecules that regulate inflammatory responses and cellular biological behavior has emerged as a promising target for the treatment of diseases associated with bone remodeling disorders.

At the initial stage of bone regeneration, the disruption of blood vessels during bone trauma leads to the formation of a hematoma, which isolates the site of injury from adequate blood perfusion, thereby exacerbating local hypoxia.^14^ Hypoxia-inducible factor-1 (HIF-1) serves as a pivotal transcriptional regulator in cellular response to hypoxia.^15^ HIF-1 consists of HIF-1α and HIF-1β subunits, which interact with the hypoxia-responsive element (HRE) to further modulate the expression of target genes. Under normoxic conditions, HIF-1α is degraded via the ubiquitination pathway mediated by proteins such as prolyl hydroxylase domain (PHD) enzymes and the von Hippel–Lindau (VHL) tumor suppressor protein.^16–18^ However, hypoxia inhibits the hydroxylation of HIF-1α, causing its accumulation in the nucleus where it binds to HIF-1β and initiates transcription of hypoxia-related genes such as vascular endothelial growth factor (VEGF).^19–22^ Therefore, the hypoxic microenvironment during bone regeneration plays a critical role in regulating HIF-1α signaling. HIF-1α is involved in the regulation of bone regeneration, which is primarily mediated through two distinct mechanisms: (1) Facilitating the synergistic interaction between angiogenesis and osteoblast activity to enhance bone formation; and (2) orchestrating metabolic adaptation in osteoblasts by upregulating anaerobic glycolysis pathways, thereby sustaining cellular bioenergetics in hypoxic microenvironments and potentiating bone repair processes.^23^

In addition to the hypoxia-mediated HIF-1α pathway, circadian rhythm also plays an important role in bone regeneration. The circadian rhythm is regulated by a group of central clock genes, among which *Brain and muscle aryl hydrocarbon receptor nuclear translocator-like protein 1 (Bmal1)* is a frequently mentioned gene in regulating bone metabolism and bone homeostasis.^24^ These genes form negative feedback loops that regulate the circadian rhythm of biological metabolism through transcription and translation processes. BMAL1 and *Circadian Locomotor Output Cycles Kaput* (CLOCK) can form a heterodimer that binds to intragenic E-boxes, stimulating the transcription and translation of genes.^25,26^ BMAL1 is the core of the circadian rhythm. Knocking out the Bmal1 gene in mice results in the immediate and complete loss of circadian rhythms in continuous darkness, providing direct evidence for its indispensable role in the generation of circadian rhythms.^27^ A significant body of research has demonstrated a close relationship between circadian rhythm and the physiological activities of bone. The circadian rhythm plays a crucial role in endochondral ossification, facilitating rapid DNA replication in cells during the day, cell mitosis, and matrix synthesis in the evening.^28^ Through its effects on key factors like osteoblast-mediated bone formation, osteoclast-driven resorption, and communication between these two cell types, circadian rhythms can modulate the balance between bone formation and resorption during bone remodeling.^28–31^

Intriguingly, many studies have shown that hypoxia and circadian rhythms interact and mutually regulate each other.^32^ The circadian rhythm influences cellular responses to hypoxia,^33^ while hypoxia can disrupt the balance of the biological clock.^34^ BMAL1 has also been identified in various cell types as a regulator of HIF-1 activity under low oxygen conditions.^35^ Current studies indicate a certain relationship between BMAL1 and HIF-1α in osteogenesis,^36^ where BMAL1 shares some structural similarities with HIF-1α.^37,38^ Under specific hypoxic conditions, BMAL1 forms a heterodimer with HIF-1α to regulate physiological processes.^35,39–41^ Above evidence indicates the crosstalk between BMAL1 and the hypoxic microenvironment in modulating bone regeneration, though the specific regulatory mechanism remains unclear. This manuscript reviews the role of HIF-1α and BMAL1 in regulating bone regeneration, discusses the crosstalk between hypoxia response and circadian rhythm in bone regeneration, and summarizes their possible role in oral and systemic bone metabolic diseases.

## HIF-1α-mediated cellular response during the inflammatory phase

### Progression of the inflammatory phase

The inflammatory phase can be further divided into two components: the formation of blood clots and the migration of inflammatory cells. For example, tooth extraction leads to rupture of blood vessels in and around the socket, resulting in immediate bleeding and local hematoma formation.^8^ Damaged blood vessels and cells release proteins that initiate the coagulation cascade, leading to clot formation.^8^ This can lead to blockage of blood supply in the wound area, resulting in hypoxia, exacerbating inflammation, acidity, and lower temperature, and is rich in calcium and lactic acid.^42,43^ Hypoxia affects various biological processes such as cell proliferation, differentiation, migration, chemotaxis, phagocytosis, and apoptosis.

Blood clots contain numerous growth factors that induce and facilitate the migration of neutrophils, monocytes/macrophages, etc., to the site of injury.^43^ According to the measurement of bone aspirates, the oxygen level reaching the bone tissue is approximately 6.6%–8.6%,^44^ while the rupture of blood vessels at the fracture site will cause further local hypoxia, with the pO2 dropping to 0.8%–3%.^45^ Following the release of inflammatory signals from local tissues at the defect site during the inflammatory stage, neutrophils are typically the first and most abundant immune cells to arrive.^46^ They subsequently orchestrate the recruitment of other immune cell types and BMSCs by releasing cytokines, thereby establishing an appropriate immune microenvironment that initiates the bone regeneration process.^47^ Simultaneously, these cells undergo differentiation and heightened activity, thereby initiating an inflammatory response aimed at removing dead cells and tissue debris from the area.^48^ Within 2–3 days following tooth extraction, a significant influx of inflammatory cells migrates to the defect site, where they merge with vascular buds and immature fibroblasts to form granulation tissue.^3^ Vascular endothelial cells also undergo extensive proliferation, giving rise to numerous new blood vessels within the matrix that facilitate oxygen and nutrient supply for an increasing number of cells.^49,50^ Then, bone marrow mesenchymal stem cells (BMSCs) accumulate, undergo proliferation, and deposit extracellular matrix within the defect area, gradually replacing blood clots.^51^ Subsequently, BMSCs surround these newly formed blood vessels and proceed to proliferate and differentiate into osteoblasts in order to directly generate woven bone.^52^ The formation of woven bone can be observed within a time frame as short as 2 weeks following tooth extraction in humans.^3,53^

**Fig. 1:**
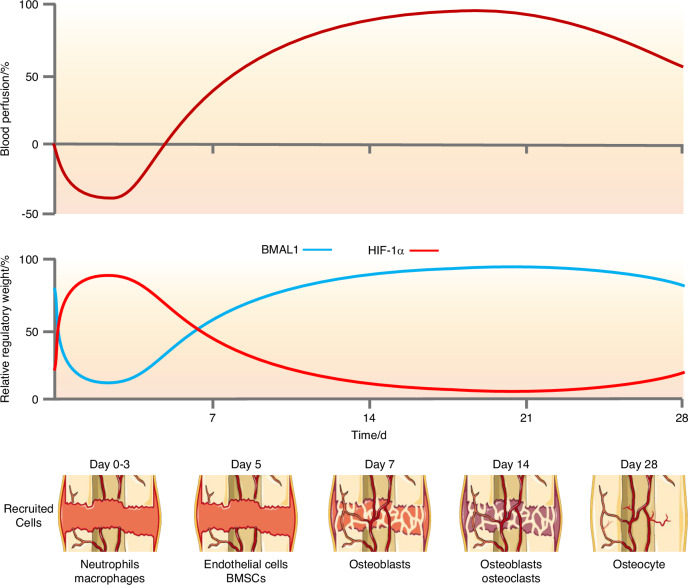
Temporal changes during bone regeneration. The blood flow in the bone defect area initially decreased due to vascular rupture and contraction. Subsequently, the blood supply gradually recovered as new blood vessels formed. The activities of HIF-1α and BMAL1 are altered. HIF-1α is activated by initial hypoxia, while BMAL1 is suppressed; both subsequently change as oxygen levels recover. The relative regulatory weight is represented as the change in the dominant role of the two in cellular regulation. The sequential appearance of different cell types is shown at the bottom

As the blood supply is established, the oxygen pressure gradually returns to normal. And the recovery of oxygen pressure is associated with the sequence in which cells appear. During bone regeneration, the level gradually decreases until it starts to rise again after about 2–3 days, and it takes 2–3 weeks to return to the highest level.^13^ Neutrophils arrive at the fracture site within hours, accompanying hematoma formation. Subsequently, by approximately 3 days, endothelial cells and immune cells such as macrophages appear. Mesenchymal stem cells are recruited by approximately 5 days, followed by the emergence of osteoblasts around 7 days. By 2 weeks, osteoblasts and osteoclasts couple to initiate the bone remodeling process.^2,3,54^ It is worth noting that, although blood perfusion has returned, these cells are still working in a hypoxic environment, and the degree of hypoxia is gradually changing. Under such challenging hypoxic conditions, each healing process initiates with a phylogenetically conserved inflammatory response. However, its spatial and temporal intensity must be strictly regulated.^9^ Pro-inflammatory signaling constitutes a critical event for launching the healing cascade and facilitates the recruitment of diverse cell types. Yet the timing of this process is paramount—if pro-inflammatory signals persist abnormally, healing becomes delayed or even arrested.^55^ Consequently, successful bone regeneration demands precise signal control to navigate the cellular challenges posed by shifting oxygen environments: the transition from normoxia (before bone defect) to hypoxia (during bone regeneration) and then back to normoxia (after bone regeneration). This regulatory mechanism may be mediated by the integration of hypoxia response and circadian rhythm. Concurrent with the restoration of oxygen tension sensed by PHDs, HIF-1α undergoes progressive degradation, leading to a declining role in cellular regulation.^16–18,56^ Meanwhile, the improved oxygen availability alleviates the hypoxic suppression of BMAL1, thereby enabling its transcriptional activity and promoting a pro-regenerative outcome in bone regeneration.^57^ A schematic of the temporal changes in blood perfusion, HIF-1α, BMAL1, and the sequence of cell recruitment is presented in Fig. 1.

### HIF-1α-mediated cellular response

#### Neutrophils

Neutrophils play both positive and negative roles in bone regeneration.^58^ Herath et al. demonstrated that local application of autologous neutrophils significantly enhanced bone regeneration in a rabbit calvarial defect model. The positive effect was particularly evident at the early healing stage (4 weeks), and repetitive application yielded superior outcomes compared to a single application.^59^ However, an excessive number of neutrophils will lead to disorders in bone regeneration.^60,61^ Uncontrolled inflammatory factors attract excessive neutrophils, which inhibit the osteogenic differentiation of BMSCs and subsequent mineralization.^62^ The formation of neutrophil extracellular traps (NETs) inhibits the migration and differentiation of MSCs, resulting in cell death and compromised bone formation.^63^ Consequently, the proper functioning of neutrophils critically influences the outcome of bone regeneration.

**Fig. 2:**
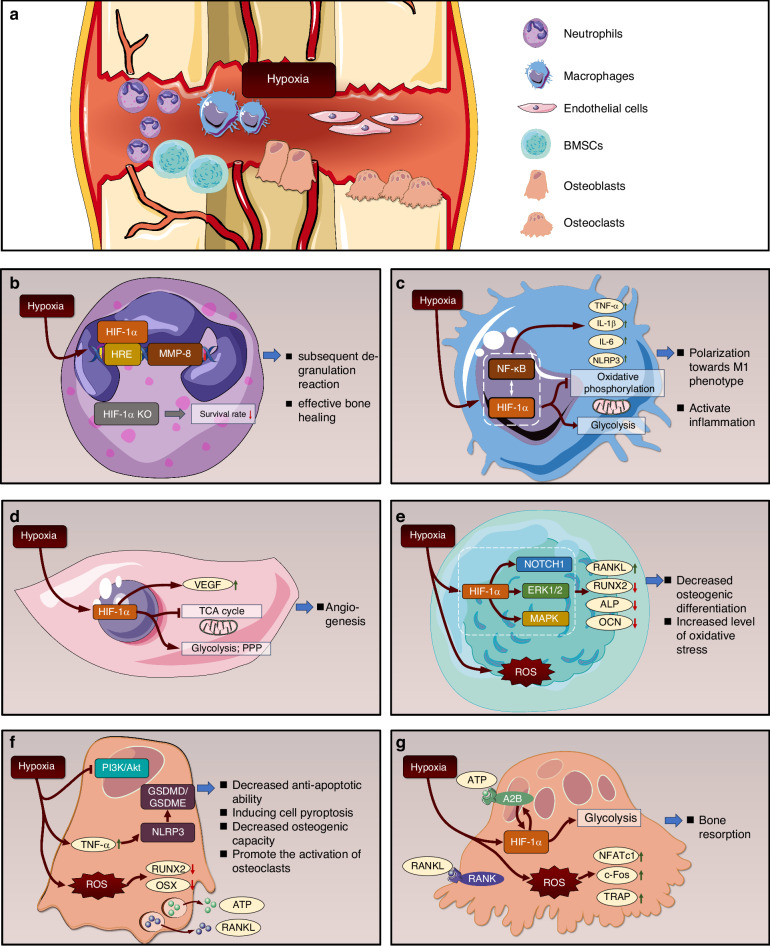
Cellular response under hypoxic conditions. **a** Six cell types—neutrophils, macrophages, endothelial cells, mesenchymal stem cells, osteoblasts, and osteoclasts—participate in bone regeneration, all of which are influenced by the hypoxic microenvironment during the inflammatory phase. **b** Under hypoxic conditions, HIF-1α binds to the HRE of MMP-8 to promote its expression, thereby facilitating the degranulation reaction of neutrophils. Knocking out HIF-1α will weaken the anti-apoptotic ability of neutrophils. **c** Under hypoxic conditions, the crosstalk between HIF-1α and NFκB pathways activates macrophage inflammatory responses and promotes M1 polarization. **d** Hypoxia-induced HIF-1α activation upregulates VEGF expression and enhances glycolysis in endothelial cells, thereby promoting angiogenesis. **e** Under hypoxic conditions, the intracellular oxidative stress level in BMSCs is significantly elevated, and their osteogenic differentiation potential is inhibited. **f** Hypoxic condition activates the TNF-α pathway within osteoblasts, inhibit the PI3K/Akt pathway, aggravate oxidative stress response, promote osteoblast apoptosis, and inhibit osteogenic ability. **g** Under hypoxic conditions, osteoclasts exhibit elevated oxidative stress levels and receive signaling molecules (ATP, RANKL) from osteoblasts, which collectively enhance intracellular glycolysis and ultimately lead to increased bone resorption activity

The initial stage of the inflammatory period of bone regeneration is an acute hypoxic microenvironment. The response of neutrophils to local oxygen tension is regulated by HIF-1α.^64^ Neutrophil degranulation is an important form in which it exerts its functions.^65^ Research has shown that hypoxia triggers a rapid degranulation of neutrophils within a short period of time (2 h),^66,67^ but this process does not seem to rely on HIF-1α at this stage.^68^ However, the subsequent degranulation reaction (lasting for 24 h) was HIF-1α-dependent, manifested by an increase in the expression of the MMP-8 gene in the promoter region that contains HREs.^67^ Furthermore, HIF-1α plays a direct role in the regulation of neutrophil apoptosis. The neutrophils isolated from the bone marrow of myeloid-targeted HIF-1α knockout mice showed a significant decrease in survival rate under hypoxic conditions (Fig. 2b).^69^ In summary, neutrophils play a balanced role in bone regeneration, where their beneficial effects are concentration-dependent. The hypoxic fracture microenvironment critically regulates their sustained functions, such as degranulation and survival, primarily through HIF-1α signaling. Thus, HIF-1α emerges as a key modulator of neutrophil activity, ensuring a controlled inflammatory response that supports effective bone healing.

#### Macrophages

During the inflammatory phase, macrophages not only phagocytose necrotic cells and tissue debris at the fracture site but also recruit mesenchymal stem cells and vascular progenitor cells from the periosteum, bone marrow, and blood circulation.^70^ Depending on their environment, macrophages can polarize their phenotype into either a pro-inflammatory (M1) or anti-inflammatory (M2) category.^71^ Under hypoxic conditions, macrophages exhibited marked upregulation in the expression of inflammatory cytokines (interleukin 1β (IL-1β), interleukin 6 (IL-6), tumor necrosis factor-α (TNF-α)) and NLRP3 inflammasome components (NLRP3, ASC, pro-caspase-1), demonstrating a pronounced inflammatory response.^72^ During the inflammatory phase of bone regeneration, acute hypoxia activates macrophage nuclear factor kappa-B (NF-κB) signaling, amplifying inflammation. This hypoxia-inflammation interplay involves HIF-1α (hypoxia regulator) and NF-κB (inflammation regulator) interaction.^73^ HIF-1α promotes IL-1β production and drives inflammation by shifting activated macrophage metabolism from oxidative phosphorylation to glycolysis, with the resulting lactate serving as a key mediator of macrophage phenotype through epigenetic and chromatin-mediated gene regulation.^74^ Ultimately, the glycolytic pathway may influence macrophage polarization toward an M1 phenotype.^71^ Consequently, within the hypoxic microenvironment during the inflammatory phase of bone regeneration, macrophages manifest potent pro-inflammatory effects mediated by HIF-1α (Fig. 2c).

While studies specifically examining the effects of myeloid HIF-1α deletion on macrophages in the context of bone regeneration are currently lacking, insights from other pathological conditions highlight its essential role in regulating macrophage survival and function. Following LPS stimulation in myeloid-specific HIF-1α knockout mice (*LysM-Cre, Hif-1α*^*flox/flox*^), Karshovska et al. observed that the absence of HIF-1α resulted in the upregulation of anti-inflammatory markers, including Mrc1 and Ym1, in macrophages.^75^ Furthermore, their investigation revealed that *Hif-1α* deletion enhanced oxidative phosphorylation, elevated ATP levels, and upregulated the expression of genes related to mitochondrial proteins. This metabolic shift consequently led to a reduction in reactive oxygen species production and necroptosis. Scheerer et al.^76^ reported that myeloid HIF-1α deficiency(*LysM-Cre, Hif-1αflox/flox*) resulted in a critical defect in clearing necrotic cell debris and regenerating endothelial networks following soft tissue injury. This defect was attributed to the delayed invasion of macrophages into the damaged site. In summary, HIF-1α is a pivotal driver of the potent pro-inflammatory response in macrophages within the hypoxic fracture microenvironment, primarily by promoting a glycolytic metabolic reprogramming. However, the specific impact of disrupting this pathway via myeloid-specific HIF-1α deletion on macrophage function and the overall bone regeneration process remains an open and critical question.

#### Endothelial cells

The development and maintenance of the functional circulatory system rely on the proliferation, migration, differentiation, and apoptosis of endothelial cells (ECs).^77^ Following the bone defect, there is a substantial increase in the proliferation of vascular endothelial cells, which promotes neovascularization and supplies oxygen and nutrients to the damaged area.^49,50^ Hypoxic microenvironment promotes intracellular VEGF expression, thereby enhancing angiogenic capacity.^78^ This occurs because hypoxia induces HIF-1α accumulation and nuclear translocation, enabling its binding to the *Vegf* gene promoter and subsequent transcriptional activation.^79^ VEGF plays a crucial role in angiogenesis by activating genes related to blood vessel formation. The expression of VEGF can be promoted by hypoxic environments, inflammation-related cytokines, and hormones.^80^ Endothelial cell-specific knockout of HIF-1α (*Tie-2-Cre, HIF-1α*^*loxP/loxP*^) disrupts a hypoxia-driven VEGF autocrine loop, leading to impaired proliferation, migration, and tube formation of endothelial cells.^81^

ECs also respond to hypoxia through metabolic alterations. Hypoxia induces metabolic reprogramming in ECs, characterized by enhanced glycolysis and pentose phosphate pathway (PPP) activity alongside reduced tricarboxylic acid (TCA) cycle flux and mitochondrial respiration. Notably, inhibition of either glycolysis or PPP (or conversely, promotion of mitochondrial respiration) reverses hypoxia’s capacity to maintain cellular stemness.^82^ Under hypoxic conditions, the forward TCA cycle decreases, while the reverse TCA cycle increases. Pharmacological inhibition of the reverse TCA cycle attenuates *Hif-1α* and *Vegf* expression, ultimately impairing ECs proliferation and angiogenic potential (Fig. 2d).^83^ Specific knockout of endothelial HIF-1α (*Tie-2-Cre, HIF-1α*^*loxP/loxP*^) downregulated Glucose transporter-1 (Glut1) expression and consequently impaired tissue glucose utilization.^84^ Together, these findings establish HIF-1α as a central hub in ECs, integrating hypoxic signaling, gene expression, and metabolic adaptation to support vascularized bone repair.

#### BMSCs

The efficacy of bone regeneration is largely determined by the cellular state of BMSCs, with varying degrees of hypoxia exerting differential effects. Several studies have investigated the impact of different oxygen concentrations on BMSCs. When cultured under moderate hypoxia (5% O₂), BMSCs exhibit suppressed senescence and enhanced osteogenic differentiation efficiency.^85–88^ However, more severe hypoxia may inhibit osteoblast growth and differentiation, ultimately impairing bone formation.^89^ Under extreme hypoxia (1%–2% O₂), the Notch1 and extracellular signal-regulated kinase 1/2 (ERK1/2)/p38 mitogen-activated protein kinase (MAPK) signaling pathways are activated in BMSCs, leading to suppressed osteogenic differentiation.^90–92^ Hypoxia reduces both the expression and activity of Runt-related transcription factor 2 (RUNX2), and the differentiation of mesenchymal cells into osteoblasts is reduced.^93^ Furthermore, hypoxia downregulates *Runx2* expression in pre-osteoblasts while promoting receptor activator of NF-κB ligand (RANKL) synthesis. Subsequent hypoxia and/or RANKL stimulation enhances tartrate-resistant acid phosphatase (TRAP) production in macrophages.^94^

The hypoxic microenvironment reduces osteogenic efficacy during bone regeneration, not only by directly reducing cellular activity but also by promoting the accumulation of pro-inflammatory mediators such as reactive oxygen species (ROS).^95^ Elevated ROS levels can induce apoptosis in both osteoblast precursor cells and mature osteoblasts, while simultaneously suppressing the expression of osteogenic markers including alkaline phosphatase (ALP), osteocalcin (OCN), and RUNX2, thereby impairing BMSC osteogenic differentiation (Fig. 2e).^96–98^ However, hypoxia-activated HIF-1α signaling may exert protective effects on BMSC function. The HIF-1α pathway enhances BMSC proliferation and migration. Pharmacological stabilization of HIF-1α using IOX2 (a prolyl hydroxylase domain inhibitor) increases intracellular Ca²⁺ while reducing ROS levels, significantly improving cellular proliferation/migration, and facilitating fracture repair.^99^ Similar pro-regenerative effects were observed with another HIF-1α-stabilizing agent, which likewise elevated BMSC proliferative and migratory capacity.^100^ HIF-1α overexpression promoted the repair of rat calvarial defects in vivo by enhancing vascularization, which was achieved through the significant upregulation of key angiogenic factors (VEGF, SDF-1, bFGF, PLGF, ANGPT1, and SCF) at both the mRNA and protein levels in BMSCs.^101^ Furthermore, overexpression of HIF-1α can also significantly enhance the osteogenic differentiation ability of BMSCs.^102^ Nevertheless, the net outcome of hypoxia on BMSCs appears to depend on the balance between its inhibitory effects—mediated partly by oxidative stress and inflammatory pathways—and the protective, pro-regenerative responses orchestrated by HIF-1α signaling. While in vitro models have limitations in fully recapitulating the in vivo hypoxic condition, current evidence suggests that strategies aimed at stabilizing HIF-1α or reducing oxidative stress can enhance BMSC function and support bone regeneration.

#### Osteoblasts

Hypoxic microenvironments delay osteoblasts’ growth and differentiation, significantly impairing bone formation capacity.^89^ This occurs through hypoxia-mediated suppression of matrix mineralization-related enzymes, as well as reduced expression and activity of alkaline phosphatase.^89^ Furthermore, hypoxia inhibits the PI3K/Akt pathway, interfering with the anti-apoptotic ability of osteoblasts.^103^ The concomitant inflammatory microenvironment under hypoxia leads to progressive accumulation of TNF-α, which induces osteoblast pyroptosis and suppresses osteogenic differentiation via activation of the NLRP3-GSDMD/GSDME pathway.^104^ This may be associated with the aggravation of inflammatory responses when cells around osteoblasts, such as macrophages, are stimulated by hypoxia. Furthermore, hypoxia stimulates osteoblasts to release adenosine triphosphate (ATP), which promotes osteoclast activation and subsequent bone resorption.^105^ Hypoxia also intensifies intracellular oxidative stress in osteoblasts, increasing ROS production and activating mitochondrial apoptotic pathways that ultimately lead to cell death.^106,107^ These ROS inhibit *Runx2* and *osterix (Osx)* expression, further diminishing osteogenic activity (Fig. 2f).^108^

However, hypoxia does not always exert detrimental effects on osteoblasts. Chronic intermittent hypobaric hypoxia, a treatment with moderate hypoxia simulating high altitude, interrupted by normoxia, may activate HIF-1α and osteogenesis-related genes to enhance fracture healing, while increasing bone mineral density and mechanical strength.^109^ Hypoxic stimulation upregulates VEGF, establishing an angiogenesis-osteogenesis coupling mechanism that concurrently promotes bone vascularization and potentiates bone formation.^56,110^ HIF-1α overexpression enhanced osteogenic differentiation, as evidenced by increased osteoblast proliferation, elevated ALP activity, and promoted nodule mineralization, potentially by inducing autophagy.^111^ Another study has shown that the hypoxic environment upregulates the expression of HIF-1a, which activates the BMP4/SMAD signaling pathway, leading to an increase in ALP content and enhanced expression of osteogenic-related factors OCN and OPN, thereby promoting the osteogenic differentiation of BMSCs.^112^ Osteoblast-specific knockout of HIF-1α (OC-Cre, HIF-1α^f/f^) severely impairs both neoangiogenesis and bone regeneration essential for skeletal repair.^113^ In summary, the hypoxic microenvironment exerts a dualistic effect on osteoblast function and bone regeneration. Acute or severe hypoxia impairs osteogenic differentiation and promotes apoptosis by inducing oxidative stress, suppressing key osteogenic pathways, and exacerbating inflammatory responses. In contrast, moderate or intermittent hypoxia can enhance osteogenic activity by activating HIF-1α, which upregulates VEGF to couple angiogenesis with osteogenesis or stimulates signaling pathways such as BMP4/SMAD. The indispensable role of HIF-1α is evident as its deficiency severely compromises bone repair, highlighting its pivotal function as a central regulator of hypoxic adaptation and a determinant of regenerative outcomes.

#### Osteoclasts

During the inflammatory phase of bone regeneration under hypoxic condition, both the number and activity of osteoclasts are significantly enhanced.^114^ Remarkably, osteoclast activity increased 21-fold in exposures to 2% oxygen.^115^ This is because the main energy driving form of osteoclasts is glycolysis.^116^ Glycolysis is the main energy source for bone resorption.^117^ Hypoxia regulates the intracellular glucose metabolism to be dominated by glycolysis, and the high glycolytic rate of osteoclasts may be further potentiated under low oxygen conditions.^118^ HIF-1α has been identified as a crucial metabolic switch that activates anaerobic respiration, rapidly boosting ATP production in osteoclasts.^119^ By upregulating the expression of pro-osteoclastic genes and enhancing glycolytic activity, HIF-1α directly stimulates bone resorption.^120^

The hypoxia-induced increase in osteoclast numbers may result from bidirectional interactions between osteoblasts and osteoclasts.^105^ On one hand, HIF-1α modulates osteoclastogenesis by regulating the RANKL/osteoprotegerin (OPG) ratio secreted by osteoblasts.^121^ On the other hand, hypoxia stimulates osteoblasts to secrete adenosine, which activates the P1 adenosine receptor A2B on osteoclast membranes. HIF stabilization enhances A2B transcription and subsequent signal transduction. This augmented A2B activation further stimulates HIF-1α through an intracellular feedback loop, ultimately amplifying glycolytic flux and mitochondrial reductase activity.^122^ HIF-1α is indispensable in osteoclasts. It can affect the absorption of calcified cartilage matrix by osteoclasts through the AMPK signaling pathway. Conditional knockout of HIF-1α in osteoclasts of mice (Ctsk-cre, HIF-1α^flox/flox^) will result in severe condylar deformity during condylar growth, with shortened condylar length and disordered fibrocartilage arrangement.^123^ Besides, HIF-1α enhances the osteoclast-mediated differentiation stimulation of BMSCs by secreting CT-1. Knocking out HIF-1α in osteoclasts inhibits bone resorption and the expression of CT-1.^124^Furthermore, the hypoxic microenvironment elevates intracellular oxidative stress in osteoclasts, leading to increased ROS production.^119^ Functioning as secondary messengers, ROS participate in RANKL signaling during monocyte-osteoclast differentiation by upregulating nuclear factor of activated T-cells cytoplasmic 1 (NFATc1) expression.^125^ ROS induction enhances the expression of key osteoclast markers, including c-Fos, NFATc1, and TRAP (Fig. 2g).^126^ Collectively, these hypoxia-mediated mechanisms potentiate osteoclast activity, resulting in enhanced bone resorptive capacity during the inflammatory phase of bone regeneration.

## Role of BMAL1 in the inflammatory phase of bone regeneration

### Role of BMAL1 in neutrophils

Neutrophil activity is subject to circadian regulation. The kinetics of neutrophil recruitment to tissues in response to damage or endotoxin challenge in mice are influenced by the zeitgeber time of the insult and correlate with the rhythmic expression of endothelial adhesion molecules.^127^ Moreover, the demonstrated circadian oscillations in the expression of cell adhesion molecules and in neutrophil phagocytic ability provide further evidence for the circadian control of neutrophil recruitment and function.^128,129^ The aging process of neutrophils themselves is also influenced by the circadian clock, as the proportion and number of aged neutrophils in the blood fluctuate throughout the day.^130^

**Fig. 3:**
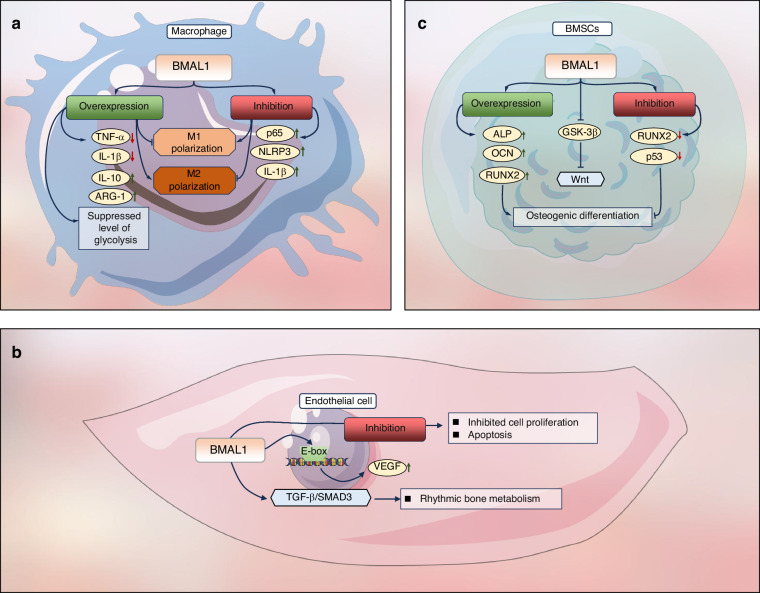
Role of Bmal1 in macrophages, endothelial cells, and BMSCs. **a** Overexpression of Bmal1 in macrophages downregulates M1-type markers (TNF-α, IL-1β) and upregulates M2-type markers (IL-10, ARG-1), while suppressing glycolytic activity to promote macrophage M2 polarization. Conversely, inhibition of Bmal1 enhances M1 polarization of macrophages. **b** In endothelial cells, BMAL1 binds to the E-box element of VEGF to promote its expression, while simultaneously activating the Transforming Growth Factor-β (TGF-β)/Small Mother Against Decapentaplegic (SMAD3) pathway, enabling endothelial cells to modulate bone tissue metabolism. Conversely, Bmal1 inhibition suppresses endothelial cell proliferation and promotes apoptosis. **c** The osteogenic differentiation capacity of BMSCs is regulated by BMAL1 expression. BMAL1 inhibits glycogen synthase kinase-3β (GSK-3β) to activate the Wnt pathway, and its overexpression upregulates osteogenic markers (ALP, OCN, RUNX2) to promote differentiation. Conversely, BMAL1 knockdown suppresses RUNX2 and p53 expression, impairing osteogenesis

The chemokine C-X-C motif chemokine ligand 2 (CXCL2), also known as macrophage inflammatory protein-2-alpha (MIP-2α), influences neutrophil recruitment and activation via MAPK signaling by binding and activating its specific receptors, CXCR1 and CXCR2.^131^ Activation of CXCR2 has been shown to promote neutrophil aging. Research has revealed that the core clock gene BMAL1 controls neutrophil aging in a cell-autonomous manner by regulating the expression of CXCL2 (Fig. 3). Genetic deletion of Bmal1 or Cxcr2 in neutrophils impedes the manifestation of cellular aging, whereas knockout of Cxcr4, a negative regulator of CXCR2 signaling, accelerates it. Neutrophil aging disrupts cytoskeletal integrity, thereby impairing neutrophil migration.^132^ Furthermore, the expression level of CXCL5 itself exhibits robust circadian oscillation and can recruit neutrophils to sites of tissue damage in a time-dependent manner.^133^ However, it has also been suggested that BMAL1 accumulation in the nuclei of neutrophils is minimal, with only a low-phosphorylation form of the protein detectable. Given that the BMAL1 function is highly dependent on its nuclear localization and phosphorylation status, the molecular oscillator governed by Bmal1 may not operate effectively in mature human peripheral neutrophils.^134^ Since the initial inflammatory phase is crucial for bone repair, the circadian clock’s control over neutrophil activity and aging presents a novel temporal mechanism that could affect the efficiency of bone regeneration.

### Role of BMAL1 in macrophages

The activity of macrophages exhibits a distinct circadian rhythm, and approximately 8%-15% of genes in macrophages display circadian oscillations.^135–137^ The transcription levels of Bmal1 in M1 macrophages show reduced amplitude and rhythmicity, but do not significantly affect the period of oscillation. On the other hand, the circadian oscillation cycle of M2 macrophages is extended without causing any changes in amplitude or rhythmicity.^138^ In Zhou et al.‘s study,^139^ overexpression of Bmal1 suppressed the expression of M1 macrophage markers (TNFα, IL-1β) while increasing the expression of M2 markers (IL-10, arginase-1 (ARG-1)) (Fig. 3a). They found that blocking the glycolysis pathway could significantly inhibit the M1 polarization, and after overexpression of Bmal1, the glycolysis level was effectively suppressed.^139^ This is also consistent with the results of Hong et al.^140^

Bmal1 regulates the inflammatory response of macrophages. Knocking out Bmal1 in macrophages can disrupt the NF-κB pathway and increase the expression of p65, thereby enhancing the expression of inflammatory factors.^141^ The NLRP3 inflammasome is the core of the inflammatory response in macrophages. It amplifies inflammation by processing and releasing inflammatory cytokines, such as IL-1β.^142^ The NLRP3 inflammasome is controlled by the circadian rhythm throughout the initiation and activation stages, and the absence of Bmal1 enhances the activity of the NLRP3 inflammasome.^143^ This regulation occurs through mitochondrial membrane potential (ΔΨm)-dependent mechanisms, as NLRP3 inflammasome activation requires ΔΨm oscillations that are intrinsically linked to circadian rhythms. Disruption of these rhythmic ΔΨm fluctuations potentiates inflammasome-triggered pyroptosis and IL-1β secretion (Fig. 3a).^143^ Besides, Bmal1 regulates time-dependent inflammatory responses following Toll-like receptor 4 (TLR4) activation by modulating enhancer activity, establishing circadian control over macrophage inflammatory output.^143^ In summary, during the process of bone regeneration, BMAL1 may influence the polarization state of macrophages and the inflammatory response, thereby affecting the outcome of bone regeneration.

### Role of BMAL1 in endothelial cells

The endothelial cell-participated bone regeneration process is regulated by various signaling and metabolic pathway networks. Among them, BMAL1 plays a crucial role. Astone et al.^144^ examined the expression levels of all circadian genes in ECs and found that the expression of these genes exhibited significant rhythmic oscillations within 24 h. Knockdown of BMAL1 affected the vitality of ECs and significantly inhibited cell proliferation. The cell cycle progression was blocked at the G0/G1 phase, and the percentage of apoptotic cells slightly increased, while the sensitivity to apoptotic stimuli was enhanced. This impaired the wound healing ability and the budding ability of ECs.^144^ Notably, ECs exhibit osteogenic regulatory functions. ECs lacking BMAL1 led to a significant decrease in BMSC proliferation, reduced osteogenic activity of BMSCs, a slight increase in osteoclasts in bone tissue, and ultimately, age-related bone loss.^145^ When ECs maintain robust circadian BMAL1 expression, SMAD3 phosphorylation and downstream TGF-β/SMAD3 signaling components display synchronized circadian oscillations, thereby preserving rhythmic bone metabolism. Conversely, BMAL1 ablation induces TGF-β/SMAD3 pathway activation continuously, leading to chronic suppression of BMSC proliferation and eventual stem cell exhaustion (Fig. 3b).^145–147^

Currently, it is widely believed that BMAL1 exerts its influence on angiogenesis through the regulation of VEGF. In the zebrafish model, it was discovered that BMAL1 can bind to crucial E-box elements in the VEGF promoter and activate its activity (Fig. 3b).^148^ Suppression of BMAL1 function inhibits Notch-dependent vascular sprouting.^148^ Mice with disrupted circadian rhythms exhibit reduced levels of VEGF in ischemic tissues and plasma, resulting in a diminished angiogenic response and impaired ability to restore blood supply.^149^ However, Astone et al. did not observe a significant downregulation of VEGF-α expression in ECs following BMAL1 knockdown; instead, they found a notable decrease in the expression of cell cycle regulators such as Cyclin A1 (CCNA1) and CDK1. Therefore, they suggested that BMAL1 may play a more pivotal role in regulating endothelial cells through cell cycle interaction. It has been observed that BMAL1 may upregulate the expression of VEGF and Angiopoietin 2 (ANG2) via the HIF-1α pathway.^36^ The knockout of the Bmal1 gene also leads to impaired angiogenesis in mice.^150^ Reduced Bmal1 expression enhances ox-LDL uptake by endothelial cells while impairing proliferation, migration, and angiogenesis.^150^ Therefore, BMAL1 plays a significant role in regulating the formation of blood vessels during the bone regeneration process by influencing endothelial cells.

### Role of BMAL1 in BMSCs

Bone formation initiates with the migration of mesenchymal stem cells into the site of the bone defect, followed by their subsequent proliferation and differentiation. Weger et al.^151^ provided evidence for the existence of a negative feedback regulatory mechanism controlling rhythm genes in BMSCs. BMAL1 can enhance the differentiation of BMSCs and augment the population of functional osteoblasts through activation of the classical Wnt signaling pathway, thereby exerting a positive regulatory effect on osteogenesis.^152–154^ The knockout of *Bmal1* in mice by Samsa et al.^155^ demonstrated a diminished capacity of BMSCs isolated from *Bmal1*^−/−^ mice to undergo osteoblastic differentiation in vitro. Concurrently, this study revealed that the absence of BMAL1 resulted in a mouse phenotype characterized by reduced bone mass, indicating the regulatory role of BMAL1 in the expression and function of genes involved in bone formation during bone development. *Bmal1* is capable of suppressing the expression of GSK-3β, consequently activating the Wnt signaling pathway.^156^ Overexpression of *Bmal1* can facilitate the expression of osteogenic markers such as ALP, OCN, and RUNX2 transcription factors, ultimately restoring the osteogenic differentiation potential of BMSCs (Fig. 3c).^157^ GSK-3β functions as an inhibitor within the Wnt signaling pathway and phosphorylates BMAL1 to maintain normal cell cycle progression. The expression levels of GSK-3β exhibit a negative correlation with those of BMAL1, suggesting that BMAL1 may serve as a key regulator within Wnt signaling pathways.^157^

In addition, the p53/p21 signaling pathway regulates the senescence and apoptosis of BMSCs. The decreased expression of *Bmal1* disrupts the balance of the sympathetic clock axis, thereby inhibiting p53 expression.^158^ The expression of *Smad*, a downstream signal transduction protein in the TGF-β superfamily, exhibits rhythmicity in BMSCs. The BMAL1:CLOCK heterodimer promotes *Smad3* expression, suggesting that circadian genes may regulate BMSCs' osteogenic differentiation through modulation of the *Smad* family.^159^ Furthermore, BMAL1 is involved in BMSCs development and ossification by regulating *Runx2* and *Osx*.^160^ However, knockout of *Bmal1* leads to disordered *Runx2* expression and loss of rhythm (Fig. 3c).^161^ Nevertheless, some studies have proposed a negative role for BMAL1 in regulating BMSC osteogenic differentiation; however, further investigation is required to elucidate the specific mechanism. These inconsistent results may be attributed to variations in mouse age selection during experiments or differences in BMSC extraction processes, or research methodologies employed.^162^

### Role of BMAL1 in osteoblasts

BMAL1 also plays a crucial role in the regulation of osteoblast proliferation and function. In vitro experiments have demonstrated the existence of the circadian system in osteoblasts.^155^ The absence of *Bmal1* leads to a reduction in osteoblast numbers and impairs terminal differentiation, resulting in morphological changes in bone trabecular and cortical structures.^155^ Clock genes have been shown to regulate bone formation in osteoblasts through leptin-dependent sympathetic signaling.^163^ Leptin-dependent sympathetic signaling promotes osteoblast proliferation by activating protein 1 (AP-1) expression. Conversely, β-adrenoceptors negatively regulate bone mass and osteoblast proliferation via *Bmal1* and *Per*.^164^ Additionally, BMAL1 may mediate cytoplasmic protein synthesis in osteoblasts through the rapamycin (mTOR) signaling pathway.^165,166^ Signals transmitted by the mTOR pathway promote mRNA translation. The promotion of mTOR effector protein kinase ribosomal S6 protein kinase 1 (S6K1) serves as an important regulator of translation, with mTOR stimulating translation through phosphorylation of S6K1/2. Furthermore, S6K1 rhythmically phosphorylates BMAL1. The phosphorylation of BMAL1 at S42 by S6K1 is essential for its interaction with the translational machinery and stimulation of protein synthesis, thereby placing BMAL1 in the context of other translation-related substrates of S6K1.^166,167^ These findings establish a direct molecular connection between BMAL1 and the mTOR pathway.

After *Bmal1* knockout, Li et al.^168^ observed impaired osteoblast differentiation and mineralization ability. They also found decreased expression of osteogenic markers and alkaline phosphatase activity, along with increased apoptosis and inflammation. Furthermore, *Bmal1* knockout promoted the phosphorylation of ERK and JNK, as well as mTOR activity, while inhibiting GSK-3β/β-catenin signaling and reducing β-catenin expression and GSK-3β phosphorylation.^168^ Consequently, upregulation of *Bmal1* expression may lead to elevated *bone morphogenetic proteins (Bmp2)* transcription through increased *Inhibitor of DNA binding 1* (*Id1*), *RUNX2*, and *Ocn* expression, effectively promoting osteoblast differentiation.^169^ However, Qian et al.^170^ demonstrated that knocking out *Bmal1* in osteoblasts resulted in an increase in trabecular bone volume but a significant decrease in cortical thickness within the femur of mice. Their data indicate that the absence of *Bmal1* can activate the BMP2/SMAD1 signaling pathway, thereby upregulating the BMP signaling cascade, to enhance the osteoblast activity within the bone trabeculae. This is because BMAL1 can exert transcriptional inhibitory effects by directly binding to the *Bmp2* promoter. Additionally, knockout of Bmal1 in osteoblasts led to enhanced osteoclast formation, resulting in increased bone resorption and ultimately leading to low bone mass.^171^ Therefore, it is evident that circadian rhythm plays a crucial role in regulating osteoblasts’ function and bone formation; however, further research is needed to elucidate the underlying mechanisms.

### Role of BMAL1 in osteoclasts

In vivo studies have demonstrated that genes associated with osteoclasts, such as cathepsin K (CTSK) and NFATc1, exhibit circadian rhythm expression in the cancellous bone of the femur,^172^ with *Bmal1* also confirmed to display rhythmic expression in osteoclasts.^173^ BMAL1 can regulate osteoclast differentiation and function through direct or indirect mechanisms. For instance, deactivating BMAL1 in RAW264.7 cells effectively promotes osteoclast differentiation, leading to osteoporosis by upregulating matrix metalloprotease 13 (MMP13) levels [149]. Knockdown of *Bmal1* in BMSCs or MC3T3-E1 cells, followed by co-culture with RAW264.7 cells, reduces OPG levels while enhancing osteoclast differentiation.^174^ Overexpression of *Bmal1* inhibits NF-κB signaling pathway activity, thereby suppressing osteoclast differentiation.^175^

BMAL1 is also involved in the communication between osteoblasts and osteoclasts. Clock genes in osteoblasts can regulate bone resorption. Expression of BMSCs after restoration of BMAL1 activity restrained the NF-κB pathway, restored the osteogenic ability of BMSCs, inhibited their ability to induce osteoclast formation, and improved bone metabolism and function.^175^ Takarada et al. proposed that Bmal1 is rhythmically expressed in osteoblasts and regulates RANKL expression by inducing 1,25(OH)2D3-dependent formation of osteoblast-dependent osteoclast.^171^ Osteoclasts derived from bone marrow macrophages remove old or broken bones under the stimulation of osteoblasts while releasing growth factors into the bone matrix, thereby recruiting and migrating BMSCs to repair areas where they differentiate into osteoblasts and contribute to new bone formation within specific regions during the process of bone regeneration.^176,177^ Thus, the circadian gene BMAL1 is a key regulator of bone remodeling during regeneration, as it directly inhibits osteoclast differentiation and function, and modulates the critical osteoblast-osteoclast crosstalk that determines the balance between bone resorption and formation.

## Crosstalk between hypoxia response and circadian rhythm

Hypoxia can lead to disruption of the biological clock and physiological imbalance.^178^ Patients with obstructive sleep apnea syndrome (OSA) or nocturnal hypoxemia (NH), both of which are characterized by recurrent hypoxia, tend to develop circadian rhythm disorders due to enhanced HIF-1α gene expression mediated by hypoxia in OSA.^179,180^ Under the combined influence of OSA-induced hypoxia and rhythm disorders, the homeostasis of bone metabolism is disrupted, resulting in a microenvironment that is unfavorable for bone maintenance and repair.^181,182^While research has demonstrated that the transcriptional response to hypoxia in tissues exhibits remarkable time-of-day dependency, which is governed by the endogenous circadian clock within the tissue.^183^ The liver-specific *Bmal1*-knockout mouse model (*Alb-Cre, Bmal1*^*fl/fl*^) confirmed that this time-dependent hypoxic response strictly relies on a functionally intact tissue-intrinsic clock, rather than being solely regulated by systemic signals.^183^ It can be seen that the hypoxic response is closely related to the circadian rhythm. The core regulators of each, HIF-1α and BMAL1, are functionally interconnected in the liver, enabling the integration of hypoxic signaling into the circadian framework and ensuring a time-of-day-dependent physiological response to hypoxia.^184^ These insights inspire the hypothesis that a similar regulatory axis may operate within the hypoxic microenvironment of bone regeneration, potentially influencing the progression of osteogenesis. In bone regeneration processes, such as fracture healing, the regenerative niche is often characterized by fluctuating oxygen tension and strong circadian rhythm of cellular activities. It is possible that the interplay between HIF-1α and BMAL1 helps coordinate the timing of critical events—including angiogenesis, osteoblast differentiation, and matrix mineralization—in a time-dependent manner. Disruption of this coupling could underlie suboptimal healing outcomes in conditions accompanied by circadian rhythm disorder or pathological hypoxia. In order to further clarify the potential interaction between the hypoxic response and the circadian rhythm during bone regeneration, we have further summarized the known and inferred interactions between HIF-1α and BMAL1 as follows.

### Heterodimerization of HIF-1α and BMAL1

**Fig. 4:**
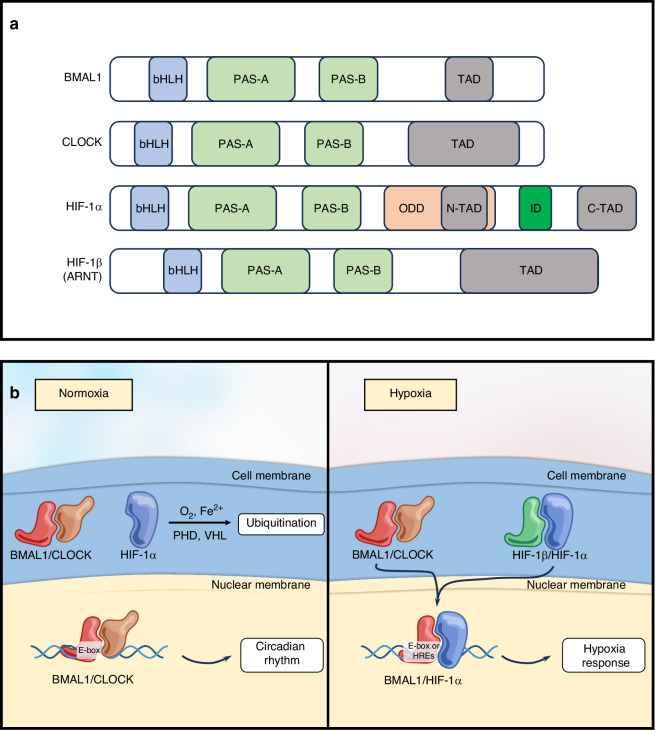
Structure of the bHLH-PAS family members and the heterodimer of BMAL1 and HIF-1α under hypoxic conditions. **a** As members of the bHLH-PAS family, BMAL1, CLOCK, HIF-1α, and HIF-1β have similar structures. **b** In normoxic conditions, BMAL1 associates with CLOCK to form a complex that translocates into the nucleus for regulating circadian-controlled genes (CCGs) transcription. In the presence of oxygen, HIF-1α is subjected to ubiquitin-mediated degradation. During hypoxia, BMAL1 has the potential to dimerize with HIF-1α and bind to E-BOX or HREs in order to initiate transcription

Currently, BMAL1 has been identified in various cell types as a regulator of HIF-1 activity under low oxygen conditions.^35,185^ This can be attributed to that BMAL1, CLOCK, HIF-1α, and HIF-1β belong to the basic helix-loop-helix-PAS (bHLH-PAS) transcription factor family, sharing structural similarities in their bHLH-PAS domains.^37,38^ The basic structures of BMAL1, CLOCK, HIF-1α, and HIF-1β all consist of: the bHLH domain that binds to DNA, the PAS domain that performs dimerization function, and the transactivation domains (TADs), and the corresponding domains between each molecule are similar (Fig. 4a).^38,186–189^ Structurally, the bHLH domain is defined by a short loop that links two α-helices (as H1 and H2), together forming a functional unit of roughly 50 amino acids.^190^ The bHLH domain is responsible for recognizing and binding to the E-box (CACGTG) DNA sequence.^188^ A PAS domain with the 260 ~ 310 amino acids is subdivided into two well-conserved PAS-A and PAS-B domains, and a loop linker.^191^ The PAS-B domain is more crucial for the dimerization of BMAL1 and CLOCK.^192^ The TAD domain interacts with transcriptional co-activators such as p300 and CBP to promote the transcription of downstream genes.^189,193^ HIF-1α contains two TADs, which are respectively called N-TAD and C-TAD. The N-TAD is associated with the degradation of HIF-1α, while the C-TAD binds transcriptional co-activators to regulate gene transcription. Additionally, HIF-1α has an oxygen-dependent degradation domain (ODDD) that overlaps with the N-TAD. This ODDD plays a crucial role in mediating the O2-regulated stability of HIF-1α.^193^

The bHLH-PAS protein family is divided into two classes based on dimerization specificity. Class I members, exemplified by the aryl hydrocarbon receptor (AhR), hypoxia-inducible factors (HIF-1α, HIF-2α, HIF-3α), and single-minded proteins (SIM1, SIM2), are unable to form homodimers or interact with other Class I partners. To become transcriptionally active, they must heterodimerize with a Class II bHLH-PAS factor. In contrast, Class II proteins readily form both homodimers and heterodimers. The archetypal Class II member is the universally expressed ARNT(HIF-1β), while other representatives include the tissue-restricted ARNT2 and the circadian regulators BMAL1 and BMAL2.^194^ In addition to the exploration of the conventional bHLH-PAS family members’ combinations, such as BMAL1/CLOCK and HIF-1α/HIF-1β, other combinations are increasingly attracting people’s interest. Among them, the combination of BMAL1 and HIF-1α is particularly worthy of study. The structure of BMAL1 shares many similarities with HIF-1β, and there are also numerous repetitions in the sequence. They exhibit sequence identities of 66% in the bHLH domain, 51% in the PAS-A domain, and 39% in the PAS-B domain.^195^ Therefore, there is a possibility of forming a heterodimer with HIF-1α. Hogenesch et al. demonstrated through yeast two-hybrid assays that HIF-1α can directly interact with BMAL1 at the protein level. Further gel shift and reporter gene assays confirmed that they form a heterodimeric complex in vitro, bind to a specific DNA response element, and drive transcription in cells while responding to hypoxic signals.^40^ This was also confirmed in the research conducted by Takahata et al.^41^ The co-immunoprecipitation experiment directly demonstrated that HIF-1α and BMAL1 have a direct physical interaction at the protein level.^196^ This indicates that they can at least form a complex or even dimerize to regulate gene transcription. The possible heterodimer formed by BMAL1 and HIF-1α can bind to the E-BOX or HRE elements of DNA chains, regulating the expression of genes such as the Circadian clock, oxidative metabolism, glycolysis, angiogenesis, etc., playing a role in integrating the hypoxic microenvironment and circadian rhythm, and may play an important role in bone regeneration (Fig. 4b).^197^

### Reciprocal regulation between HIF-1α and BMAL1

In Suyama et al.‘s experiments,^198^ no direct interaction between BMAL1:CLOCK and HIF-1α was observed; however, knockout of global *Bmal1* (*Global Bmal1*^−^^*/*^^−^) resulted in a decline in HRE activity. This suggests that BMAL1 may regulate the function of HIF-1α. They proposed that the interaction between BMAL1:CLOCK and E-box sequences in gene promoters could potentially modulate the recruitment and activity of HIF-1α at HRE sites located near or partially overlapping with E-box sequences, thereby effectively regulating the expression of its target genes.^198^ In the study conducted by Peek et al., it was similarly observed that knockdown of *Bmal1* (*ACTA-rtTA-TRE-Cre, Bmal*^*fx/fx*^) in mouse skeletal muscle resulted in a reduction in HIF-1α accumulation and decreased sensitivity to the simulated hypoxic environment. The diminished functionality of HIF-1α may be attributed to impaired transcription of Hif-1α caused by Bmal1 gene knockout. This phenomenon can potentially be explained by a reduced interaction between HIF-1α and BMAL1, leading to decreased stability of monomeric HIF-1α, as seen in cells lacking HIF-1β.^195^ However, it is also possible that BMAL1 is involved in the transcriptional regulation of the HIF-1α gene. Studies have demonstrated that BMAL1 overexpression in primary glioma cells upregulates ANG2 and VEGF expression via the HIF-1α pathway, thereby enhancing angiogenesis.^36^ Conversely, BMAL1 knockout in chondrocytes (*Col2α1-CreER, Bmal1*^*flox/flox*^) significantly reduces VEGF and HIF-1α levels while increasing MMP13 and Runx2 expression, ultimately suppressing cell proliferation and promoting apoptosis.^199^

HIF-1α is also involved in regulating the expression of BMAL1. Transfecting the expression plasmid of HIF-1α into liver cancer cells, or treating with CoCl_2_ to increase the protein level of HIF-1α in the cells, both will lead to an increase in the mRNA expression levels of *Clock* and *Bmal1*.^200^ This result is also applicable in human colorectal cancer cell lines, and they considered that this cascade promoted glycolytic activity and reduced apoptosis in hypoxic conditions.^201^ When U2OS cells were treated with HIF-1α stabilizers such as DMOG, Ni^2+^, and Co^2+^, it was observed that the rhythmic cycle of the BMAL1:LUC reporter gene was significantly prolonged, and the amplitude was weakened.^196^ In summary, a reciprocal regulatory loop exists between BMAL1 and HIF-1α, wherein BMAL1 modulates both the activity and transcription of HIF-1α, while HIF-1α in turn regulates BMAL1 expression and circadian rhythm. Although similar studies have not yet been conducted in bone tissue or bone defect models, it is noteworthy that both circadian rhythms and hypoxia signaling play essential roles in bone regeneration—orchestrating angiogenesis, cell differentiation, and metabolic adaptation. This reciprocal regulatory interplay may provide a mechanistic basis for understanding the integration of hypoxic responses and circadian rhythm during bone regeneration.

### HIF-1α and BMAL1 co-regulate intracellular oxidative stress levels under hypoxic conditions

Hypoxia can diminish the osteogenic effect during bone regeneration and proliferation due to its direct suppression of cell activity, as well as its promotion of pro-inflammatory mediators such as ROS.^95^ Elevated levels of ROS can induce apoptosis in osteoblast precursor cells and mature osteoblasts, while also interfering with the osteogenic differentiation of BMSCs by inhibiting the expression of key markers like ALP, OCN, and RUNX2.^96–98^ Therefore, ensuring a proper oxygen supply and effective removal of ROS during bone regeneration and proliferation is crucial for promoting successful bone tissue regeneration. The relationship between ROS and HIF-1α exhibits multifaceted complexity. On one hand, ROS can activate the HIF-1α signaling pathway, thereby inducing oxidative stress-related damage.^202^ Conversely, HIF-1α enhances ROS production through mechanisms such as elevating NADPH oxidase activity.^203^ Simultaneously, downstream proteins activated by HIF-1α mediate metabolic reprogramming from oxidative phosphorylation to aerobic glycolysis, while maintaining redox homeostasis. This transition reduces mitochondrial ROS generation by suppressing glucose oxidation in the TCA cycle. Furthermore, HIF-1α signaling promotes glutathione biosynthesis to bolster cellular antioxidant defense capacity, establishing a regulatory feedback loop between oxidative stress and hypoxic adaptation.^204,205^

**Fig. 5:**
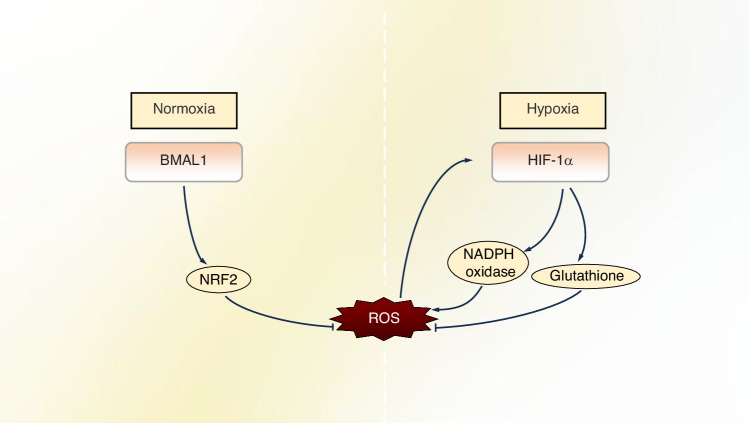
BMAL1 and HIF-1α cooperatively regulate intracellular ROS levels. BMAL1 suppresses ROS generation by upregulating the expression of NRF2, a core regulator of cellular antioxidant defense. HIF-1α can be activated by either hypoxic conditions or elevated ROS levels. HIF-1α exhibits dual regulation of ROS: (1) it increases ROS production via NADPH oxidase upregulation, and (2) it reduces ROS levels by enhancing glutathione synthesis

As a pivotal circadian rhythm gene, Bmal1 plays a central role in counteracting oxidative stress.^206^ BMAL1 orchestrates the circadian oscillation of the antioxidant transcription factor Nuclear factor erythroid 2-related factor 2 (Nrf2) expression, which governs intracellular ROS homeostasis (Fig. 5).^207^ Macrophages deficient in BMAL1 (*Lyz2Cre, Bmal1*^*LoxP/LoxP*^) exhibited a significant elevation in oxidative stress response, accompanied by the accumulation of ROS and HIF-1α, as well as a reduction in NRF2 activity.^208^ These antioxidant response pathways inhibit HIF-1α, a pivotal regulator of glycolytic metabolism and inflammation, in a ROS-dependent manner. Recent studies have demonstrated that BMAL1’s role extends beyond regulating and oscillating core clock genes to include maintaining redox balance and enhancing cell viability under oxidative conditions.^209,210^ Sun et al.‘s experiment showed that a consistent supply of oxygen can significantly increase BMAL1 levels in hypoxic microenvironments for osteoblasts.^57^ Their study also found that sustained local release of oxygen promotes rapid blood vessel formation at bone defect sites, which is beneficial for osteogenesis. Furthermore, as mentioned earlier, under certain circumstances, low oxygen conditions lead to dimer formation between BMAL1 and HIF-1α, which then bind with HREs for transcriptional activation. Thus, BMAL1 acts as a rheostat, fine-tuning HIF-1α activity to maintain redox equilibrium. Further investigation into the crosstalk between BMAL1 and HIF-1α interaction under hypoxia is crucial. Understanding this relationship is key to elucidating the mechanisms of bone regeneration.

In summary, BMAL1 and HIF-1α form a critical regulatory axis that maintains intracellular redox homeostasis under hypoxia, a key determinant for successful bone regeneration. BMAL1 counteracts oxidative stress by orchestrating the circadian expression of the antioxidant master regulator NRF2. Simultaneously, it fine-tunes the dual role of HIF-1α, which can both induce ROS production and initiate protective metabolic reprogramming to limit mitochondrial ROS generation. This cooperative regulation ensures that the hypoxic response is precisely controlled, preventing the detrimental accumulation of ROS that impairs osteoblast survival and BMSC differentiation. Therefore, the BMAL1-HIF-1α interplay is fundamental for creating a redox-balanced microenvironment conducive to bone healing.

### HIF-1α and BMAL1 co-regulate metabolic reprogramming under hypoxic conditions

With the progression of research, deeper mechanisms underlying oxidative stress regulation have been revealed, particularly the metabolic interplay orchestrated by BMAL1 and HIF-1α. These two factors jointly regulate cellular metabolic reprogramming under hypoxic conditions, which critically influences bone regeneration processes. The functional crosstalk between BMAL1 and HIF-1α fundamentally hinges on metabolic adaptation. BMAL1 and HIF-1α serve as pivotal molecular hubs integrating circadian rhythms and hypoxic adaptation to coordinate bone regeneration. Their functional interplay encompasses transcriptional regulation, oxidative stress modulation, and metabolic reprogramming, profoundly influencing cellular homeostasis and tissue repair. The BMAL1-HIF-1α axis critically governs redox balance during bone regeneration. BMAL1 maintains mitochondrial oxidative phosphorylation (OXPHOS) to sustain ATP production in human embryonic stem cell-derived cardiomyocytes, and using the CRISPR/Cas9 technology to knockout the Bmal1 gene leads to a significant weakening of mitochondrial OXPHOS and impaired cardiomyocyte function.^211^ Consequently, BMAL1 deficiency elevates ROS levels, exacerbates HIF-1α-driven inflammatory responses (e.g., IL-1β upregulation), and compromises mitochondrial function.^208,212^ In contrast, HIF-1α promotes glycolytic metabolism and reduces mitochondrial ROS generation by suppressing TCA cycle activity.^204^ This will enhance the tolerance of mitochondria to hypoxic injury, indicating that hypoxia regulates energy metabolism by adaptively inducing high expression of HIF-1α.^213^ However, HIF-1α-driven glycolytic activation promotes osteoclast-mediated bone resorption, ultimately hindering bone regeneration.^203,214^

**Fig. 6:**
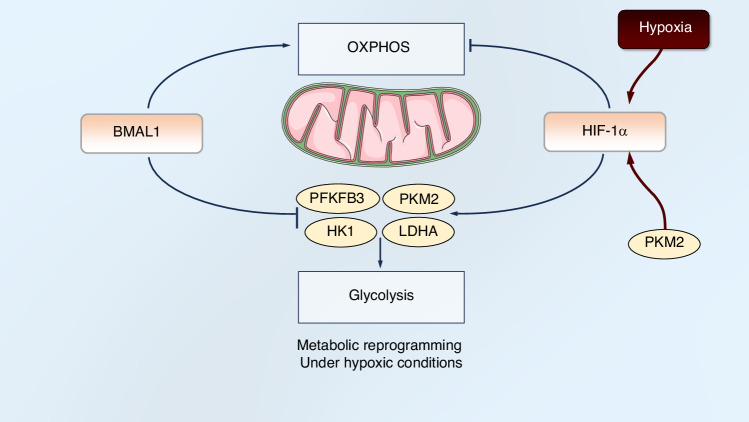
Metabolic reprogramming under hypoxic conditions is coordinated by BMAL1 and HIF-1α. Under hypoxic conditions, the HIF-1α-mediated metabolic reprogramming (suppressing oxidative phosphorylation/promoting glycolysis) can be counterbalanced by BMAL1, which restores mitochondrial oxidative phosphorylation and suppresses key glycolytic enzymes (e.g., PFKFB3, PKM2, HK1, LDHA) to constrain glycolytic flux

Some key glycolysis-related enzymes are simultaneously regulated by BMAL1 and HIF-1α. Skeletal muscle BMAL1 modulates HIF-1α-regulated glycolytic enzymes (e.g., 6-phosphofructo-2-kinase/fructose-2,6-biphosphatase 3, PFKFB3), thereby influencing glycolytic flux.^215^ PFKFB3 is recognized as a critical glycolytic regulator. In *Bmal1*-knockout mice (*HSA-rtTA;TRE-Cre, Bmal1*^*fx/fx*^), expression of *Pfkfb3* is downregulated, while subsequent VHL knockout stabilizes HIF-1α and restores *Pfkfb3* transcription and protein expression.^215^ Pyruvate kinase M2 (PKM2) serves as a rate-limiting enzyme in glycolysis, converting phosphoenolpyruvic acid (PEP) to pyruvate. PKM2 responds to lipopolysaccharide (LPS) stimulation via HIF-1α and acts as a key determinant of macrophage metabolic reprogramming, with a positive feedback loop existing between PKM2 and HIF-1α.^216^ PKM2 functions both as an activating cofactor and a transcriptional target of HIF-1α.^217^ After nuclear translocation, PKM2 directly interacts with HIF-1α to mediate mitochondrial apoptosis, leading to excessive mitochondrial ROS generation, reduced ΔΨm (mitochondrial membrane potential), disrupted mitochondrial morphology, and mtDNA release.^218^ As a co-activator for HIF target genes, PKM2 plays a role in the transformation from oxidative metabolism to glycolytic metabolism.^217^ However, BMAL1 can inhibit the transcription of PKM2, and macrophages lacking Bmal1 exhibit higher levels of glycolytic metabolism.^219^ BMAL1 overexpression inhibits glycolytic activity to suppress M1 macrophage polarization.^139^ BMAL1 regulates the level and localization of glycolysis enzyme PKM2. The knockout of Bmal1 leads to an increase in the phosphorylation of signal transducer and activator of transcription 3 (STAT3), further driving the expression of IL-1β mRNA, which may be mediated by the elevated PKM2 level.^220^ Thus, BMAL1 and HIF-1α establish functional crosstalk through PKM2. Succinate, a TCA cycle metabolite, accumulates to promote SDH-derived mitochondrial ROS production, which inhibits HIF-1α prolyl hydroxylases and stabilizes HIF-1α.^221^ The succinate-HIF-1α axis constitutes a critical link between oxidative and glycolytic metabolism: succinate oxidation promotes HIF-1α stabilization and drives glycolytic switching.^222^ Notably, succinate levels increase in Bmal1-knockout macrophages.^220^ This reveals an additional metabolic linkage between BMAL1 and HIF-1α via succinate regulation. Hexokinase 1 (HK1) catalyzes the first step of glycolysis,^223^ while lactate dehydrogenase A (LDHA) converts pyruvate to lactate in the final step of anaerobic glycolysis.^224^ Both LDHA and HK1 are transcriptionally regulated by HIF-1α.^225,226^ BMAL1 overexpression significantly suppresses cellular glycolysis and lactate production by reducing HK1 and LDHA protein levels.^227^ We have mapped the regulatory network of BMAL1 and HIF-1α in glucose metabolism as Fig. 6. Additionally, other key metabolic enzymes or metabolites regulated by BMAL1 and HIF-1α have been compiled in Table 1. Collectively, these findings demonstrate the interplay between BMAL1 and HIF-1α in metabolic reprogramming.

**Table 1:** Key metabolic enzymes/metabolites regulated by HIF-1α and BMAL1

Metabolic pathway	Target Gene / substance	Function	HIF-1α			BMAL1			
			Direction of regulation		Ref.	Direction of regulation			Ref.
Glucose metabolism	Glucose-6-phosphate dehydrogenase (G6PD)	Catalyzes the first step in the pentose phosphate pathway	HIF-1α increases G6PD mRNA level and activity	^279^			BMAL1 deficiency reduces G6PD mRNA levels	^280^	
	Pyruvate dehydrogenase kinase (PDK)	Inhibit pyruvate dehydrogenase (PDH) to convert pyruvate into acetyl coenzyme A	HIF-1α induces PDK1 expression	^281^			BMAL1 promotes PDK1 phosphorylation	^282,283^	
	Glucose transporter (GLUT)	Glucose uptake	HIF-1α significantly increases GLUT1 protein expression levels	^284^			Ablation of Bmal1 reduces GLUT4 levels	^283^	
Amino acid metabolism	Glutamine	An essential amino acid	HIF-1α suppresses the hydrolysis of glutamine	^285^			Glutamine is rhythmically regulated by Bmal1	^286^	
	Glutaminase	Catalyzes the breakdown of glutamine into glutamate and ammonia	HIF-1α induces substantial glutaminase 2 expression	^287^			Glutaminase shows rhythmic expression	^286^	
	Alanine Aminotransferase (ALT)	Transfer the amino group (-NH₂) of alanine to α-ketoglutarate, generating pyruvic acid and glutamic acid.	Stabilized HIF-1α treatment is associated with lower ALT levels	^288^			Bmal1 knockout reduces serum ALT levels	^289^	
Lipid metabolism	3-Hydroxy-3-Methylglutaryl-CoA Reductase (HMGCR)	Cholesterol synthesis rate-limiting enzyme	HIF-1α induces the ubiquitination and degradation of HMGCR	^290^			Bmal1 knockout increases expression of the Hmgcr gene	^291^	
	Fatty acid synthase (FASN)	Cellular fatty acid biosynthesis	HIF-1α upregulates the expression of FASN	^292^			BMAL1 inhibits the expression and activity of FASN	^293^	

**Fig. 7:**
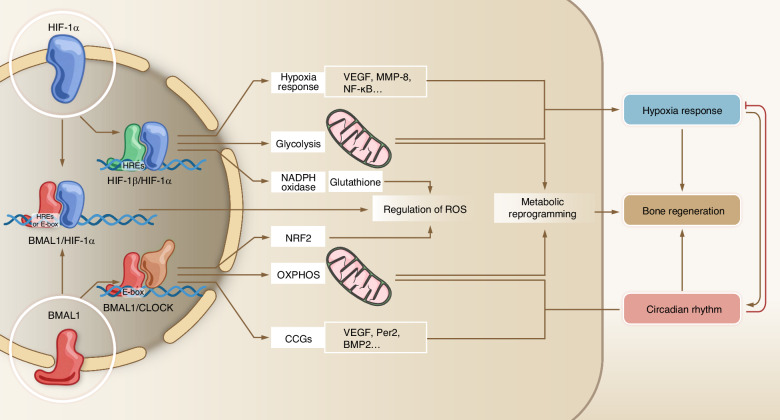
The crosstalk between BMAL1 and HIF-1α at different levels. BMAL1 /CLOCK and HIF-1α/HIF-1β bind to specific E-box and HREs, respectively. In addition, there is an interaction between BMAL1 and HIF-1α proteins. These two proteins may combine to form a heterodimer, which further regulates the cellular functions under hypoxic conditions. They co-regulate a set of downstream target genes involved in different processes. This collaborative regulation influences critical cellular functions, including the regulation of ROS and metabolic reprogramming, thereby integrating circadian rhythm and hypoxia response signals to promote bone regeneration

In summary, BMAL1 and HIF-1α co-regulate metabolic reprogramming under hypoxia by targeting key glycolytic enzymes, constituting a fundamental mechanism for bone regeneration. BMAL1 promotes oxidative phosphorylation to maintain energy supply and mitochondrial integrity, while HIF-1α drives glycolytic flux. Their interplay is exemplified through shared targets: BMAL1 inhibits PKM2, HK1, and LDHA to restrain glycolysis, whereas HIF-1α activates them. This balance is crucial, as unchecked HIF-1α-driven glycolysis can promote bone resorption. Furthermore, metabolic intermediates like succinate link BMAL1 deficiency to HIF-1α stabilization. Thus, the BMAL1-HIF-1α axis ensures a metabolically balanced microenvironment essential for effective bone repair. We summarize the crosstalk between BMAL1 and HIF-1α at different levels and their effects on bone regeneration in Fig. 7.

### HIF-2α and its interaction with BMAL1

To facilitate a more comprehensive discussion of the crosstalk between hypoxia and circadian rhythms, it is also essential to consider another well-studied key molecule in the hypoxic response: HIF-2α. HIF-2α is another isoform of HIF-α. It retains highly conserved sequences, shares similar domain structures with HIF-1α, and binds to the same HRE sequence, 5′-(A/G)CGTG-3′, in the promoters of specific target genes.^228^ While they share similar regulatory roles in bone regeneration, there are distinct differences between them. Holmquist-Mengelbier et al. found that HIF-1α is associated with the acute hypoxic response, while HIF-2α is linked to the chronic hypoxic response.^229^ In contrast to HIF-1α, which supports chondrocyte phenotype maintenance and metabolic adaptation to hypoxia, HIF-2α promotes the progression of osteoarthritis.^230^ Lee et al. found that HIF-1α and HIF-2α have similar but mechanistically distinct roles in bone remodeling: both inhibit osteoblast differentiation via the TWIST2-RUNX2-OCN axis, but HIF-1α indirectly promotes osteoclastogenesis by upregulating HIF-2α, whereas HIF-2α directly drives this process.^231^ HIF-1α and HIF-2α are involved in regulating angiogenesis (e.g., VEGF) to couple osteogenesis, but HIF-1α primarily influences early osteoblast differentiation and may inhibit load-induced bone formation, whereas HIF-2α has a more pronounced role in regulating bone mass under specific conditions (e.g., in mature osteoblasts) and may exhibit functional complementarity or specific target gene regulation compared to HIF-1α.^45^

Although there are few existing studies on the relationship between HIF-2α and BMAL1, we can still infer that there is a close connection between them. In contrast to normoxia, where HIF-2α mRNA expression is arrhythmic in zebrafish, hypoxia induces a circadian rhythm in its expression.^232^ Due to the high structural similarity between HIF-1α and HIF-2α, HIF-2α may also form a heterodimer with BMAL1. This specific dimeric complex (HIF-2α/BMAL1) may preferentially bind to gene regulatory regions containing E-box elements, thereby directly integrating hypoxic signaling into circadian rhythm regulation. A recent study has substantiated this perspective. Ruan et al. employed a combination of AlphaFold structural prediction and cryo-EM to elucidate, for the first time, the three-dimensional structure of the BMAL1/HIF2A/DNA complex. This heterodimer recognizes and binds to a non-canonical HRE sequence (CAGGTG), thereby cooperatively activating the rhythmic transcription of the downstream target gene amphiregulin (AREG) and mediating circadian-dependent cardioprotection. Knockout of *Bmal1*, *Hif-2α*, or *Areg* was sufficient to abolish the diurnal variation in myocardial infarction, establishing the central role of the BMAL1–HIF2A–AREG signaling axis.^233^ HIF-2α may also be involved in regulating Rev-erbα within the circadian rhythm system, thereby influencing BMAL1.^234^ Consequently, HIF-2α and BMAL1 are also intricately connected. However, the functional significance of this relationship in bone regeneration necessitates further elucidation.

## The clinical significance of the crosstalk between hypoxia and circadian regulation

In the preceding part, we have summarized the roles of BMAL1 and HIF-1α in bone regeneration. Clinically, numerous bone-related pathologies exhibit microenvironments analogous to those in bone defects. Under such conditions, the crosstalk between BMAL1 and HIF-1α critically influences both bone regeneration and therapeutic outcomes. Herein, we present several representative scenarios:

### Osteoporosis

#### Shift work-induced osteoporosis

A follow-up study of night-shift nurses revealed that prolonged shift work may lead to lower trabecular and cortical bone mineral density (BMD), thereby increasing the risk of hip and wrist fractures. These findings collectively indicate that night-shift work constitutes a risk factor for osteoporosis.^235,236^ Similar adverse effects on bone tissue were observed among steel workers, where night-shift workers exhibited a higher prevalence of abnormal BMD compared to day-shift workers. This prevalence increased with cumulative night-shift exposure.^237^ Night-shift work disrupts sleep patterns and impairs melatonin secretion, resulting in circadian rhythm disruption. Additionally, reduced sunlight exposure during night shifts may decrease endogenous vitamin D synthesis, further compromising skeletal health^237,238^

Circadian rhythm disruption also manifests through altered oxidative stress levels. Shift work disrupts the pro-/antioxidant balance, favoring oxidative stress and subsequent physiological dysfunction.^239^ Night-shift work can induce DNA oxidative damage, which remains elevated even after one month of routine shift work.^240^ These studies suggest that osteoporosis associated with circadian rhythm disruption may involve oxidative stress mechanisms. In conclusion, the disruption of circadian rhythms caused by shift work may disrupt the normal expression of BMAL1, thereby interfering with its synergistic interaction with HIF-1α. As mentioned earlier, BMAL1 is a key regulatory factor for maintaining the balance of intracellular oxidative stress. The loss of BMAL1 function leads to the accumulation of ROS and abnormal stability of HIF-1α protein, thereby triggering excessive inflammatory responses and damaging mitochondrial function. In the context of bone regeneration, this dysregulation of the BMAL1-HIF-1α axis will disrupt the microenvironment necessary for bone regeneration, ultimately hindering bone formation and repair processes, providing a potential mechanism explanation for osteoporosis related to shift work. Further investigation is warranted to elucidate these relationships and develop strategies for preserving bone health in shift workers.

#### Circadian rhythm disruption in diabetic osteoporosis

Diabetes mellitus (DM) is a widely prevalent chronic metabolic disease, with type 2 diabetes (T2DM) being the main type.^241^ A cohort study has shown that the prevalence of bone loss and osteoporosis in T2DM patients is 50.6% and 17.7% respectively.^242^ Hyperglycemia inhibits the maturation/differentiation of osteoblasts, accelerates the aging and apoptosis of bone cells, and exacerbates cellular oxidative stress. Concurrently, it stimulates osteoclasts, enhancing bone resorption and disrupting bone remodeling balance.^243^

Physiological glucose homeostasis is regulated by circadian rhythms. Core metabolic organs – including skeletal muscle, liver, white adipose tissue, and pancreatic islets – exhibit robust intrinsic circadian oscillations. Circadian rhythm disruption may induce insulin resistance, impair glucose homeostasis, and promote T2DM pathogenesis.^244^ Conversely, T2DM itself causes cellular circadian rhythm disruption. Research conducted by Li et al.^245^ indicates that type II diabetes in rats leads to reduced expression of the rhythm *Bmal1* gene in BMSCs. Overexpression of *Bmal1* rescues osteogenic differentiation potential of BMSCs under T2DM microenvironments. Animal studies demonstrate that eldecalcitol (ED-71), an active vitamin D analog, upregulates *Bmal1*, suppresses oxidative stress, promotes osteogenesis, and attenuates T2DM-mediated bone loss.^246^ These results strongly suggest that the downregulation of *Bmal1* in the diabetic microenvironment is a crucial factor leading to bone regeneration disorders.

In diabetes, multiple tissues are in a state of hypoxia. However, HIF-1α activation within cells fails to reach maximal levels due to impaired signaling caused by hyperglycemia and elevated fatty acids. This suppresses HIF-1α stability and function, resulting in inadequate adaptive responses to hypoxia.^247^ Suppressed HIF-1 signaling predisposes individuals to complications, including impaired wound healing, diabetic nephropathy, cardiovascular disease, diabetic retinopathy, obesity, and β-cell dysfunction.^247^ Both circadian disruption and diabetes induce aberrant *Bmal1* expression. This genetic disturbance impairs BMAL1-HIF-1α crosstalk, further compromising HIF-1α function.^195^ BMAL1 deficiency reduces glucose utilization in skeletal muscle, as BMAL1 and HIF-1α cooperatively regulate the key glycolytic enzyme PFKFB3. Notably, stabilizing HIF-1α improves impaired glucose tolerance in Bmal1-knockout mice.^215^ Therefore, in diabetic osteoporosis, factors such as high blood sugar jointly cause abnormal expression of BMAL1 and inhibition of HIF-1α function, disrupting the crucial BMAL1-HIF-1α communication. The failure of this axis directly damages multiple key processes essential for bone regeneration: the dysfunction of HIF-1α will weaken angiogenesis, while the coordinated regulation of metabolic reprogramming and the balance of oxidative stress by BMAL1-HIF-1α are also disrupted. Ultimately, the osteogenic differentiation ability of BMSCs is impaired, the balance between bone formation and bone resorption is disrupted, and bone regeneration fails. By regulating BMAL1 through drugs or genetic means to restore its normal interaction with HIF-1α, it is expected to become a new strategy for reversing diabetic bone loss.

### Osteoarthritis

Osteoarthritis (OA) is a chronic degenerative joint disorder characterized by progressive cartilage degradation, synovial inflammation, and subchondral bone remodeling, representing one of the most prevalent joint diseases.^248^ Circadian rhythm disruption is one of the key risk factors for OA.^249^ Substantial evidence indicates altered circadian rhythms in OA chondrocytes, manifesting as dysregulated clock gene expression.^250–253^ Specifically, *Bmal1* expression is downregulated. *Bmal1* knockout reduces cartilage matrix synthesis, diminishes chondrocyte proliferation, and enhances chondrocyte hypertrophy and apoptosis in condylar cartilage, ultimately inducing temporomandibular joint OA.^254^ The pathogenic effects of *Bmal1* deficiency likely involve dysregulation of TGF-β and NFAT signaling pathways, coupled with reduced expression of critical matrix-associated genes, including *SRY-box transcription factor 9 (Sox9)*, *aggrecan (Acan)*, and *Col2a1*. This disrupts metabolic signaling, exacerbates inflammation, and promotes progressive OA-like cartilage degeneration.^255^ Alternatively, *Bmal1* loss may hyperactivate the mTORC1 pathway in OA cartilage, driving cartilage degradation and chondrocyte apoptosis.^256^

HIF-1α is essential for cartilage development and homeostasis. Due to joint microstructure, chondrocytes exist in a chronically hypoxic microenvironment.^257^ Under such conditions, HIF-1α activation sustains anaerobic glycolysis and promotes extracellular matrix synthesis.^258^ However, only stabilized HIF-1α exerts protective effects.^259^ Notably, HIF-1α levels in articular cartilage and synovial fluid positively correlate with disease severity in knee OA patients.^260^ And specific knockout of HIF-1α increased the expression of MMP13 and cartilage destruction in OA mice.^261^ Cartilage degeneration coincides with disrupted hypoxia and HIF-1α degradation. Maintaining intra-cartilaginous hypoxia and stabilizing chondrocytic HIF-1α may decelerate OA progression.^262^

Crucially, HIF-1α in cartilage is regulated by BMAL1. BMAL1 not only governs circadian gene expression in cartilage but also modulates physiological chondrogenesis through coupling with HIF-1α and VEGF. After knocking out *Bmal1*, growth plate chondrocytes exhibit reduced VEGF and HIF-1α alongside elevated *Mmp13* and *Runx2* expression.^199^ Knocking out *Bmal1* in primary cultured chondrocytes also affects their anti-apoptotic ability, partly due to the inhibition of *Hif-1α* and *Vegf* expression, while HIF-1α can regulate pro-apoptotic and anti-apoptotic genes.^230,263^ While HIF-1α-mediated signaling couples with circadian rhythm in chondrocytes, the precise mechanisms underlying this interaction remain incompletely understood.^264^ Consequently, the downregulation of BMAL1 during OA disrupts the protective BMAL1-HIF-1α axis. This disruption not only leads to metabolic abnormalities, such as dysregulated glycolysis and exacerbated inflammation, but, more importantly, impairs the HIF-1α-mediated adaptive survival response of chondrocytes. This accelerates the degradation of the cartilage matrix and chondrocyte apoptosis, thereby driving OA progression. Targeting the restoration of the BMAL1-HIF-1α axis function in chondrocytes may present a novel therapeutic strategy for delaying cartilage degeneration.

### Titanium implant

Titanium is widely used in dental and orthopedic surgeries due to its mechanical properties, corrosion resistance, and biocompatibility.^265^ Implant placement disrupts local bone architecture, initiating a bone repair process analogous to alveolar bone regeneration in extraction sockets, ultimately achieving osseointegration.^266^ Circadian rhythms also participate in peri-implant osseointegration.^267^ BMAL1 overexpression in BMSCs surrounding implants enhances osseointegration.^268^ Post-implantation, surgical trauma, and persistent titanium surface oxidation create a local hypoxic microenvironment.^269^ Both BMAL1 and HIF-1α contain bHLH-PAS domains enabling hypoxia sensing. BMAL1 may heterodimerize with HIF-1α under hypoxia, activating pathways that promote osteogenic differentiation.^269^ While moderate hypoxia facilitates HIF-1α activation and peri-implant angiogenesis,^270^ excessive hypoxia (e.g., high-altitude conditions) impedes osseointegration.^271^ Recent surface modification strategies incorporate Co^2+^/Mg^2+^ ions to activate HIF-1α signaling, enhancing angiogenesis and accelerating osseointegration.^272^ It is worth noting that the surface modification of the implant also affects the peripheral circadian rhythm of human BMSCs in vitro.^273^ Here, we propose a feasible but unexplored direction: modifying the implant surface to promote the activation of BMAL1, in order to utilize the communication and interaction characteristics between BMAL1 and HIF-1α to further promote bone integration. By specifically activating BMAL1 through implant surface modification, it is possible to reinforce the BMAL1-HIF-1α signaling pathway in the postoperative hypoxic microenvironment. This enhancement synergistically improves the osteogenic capability of BMSCs and promotes angiogenesis, which are critical for successful osseointegration that requires efficient bone regeneration involving BMSC recruitment, differentiation, and new vessel formation. Consequently, this approach can accelerate and enhance the osseointegration outcome.

## Current research limitations and future research prospects

The research on the crosstalk between BMAL1 and HIF-1α has been ongoing for a long time. Many scholars have devoted their time and energy to this field and have achieved significant results. However, when summarizing the existing research, there are still some shortcomings. Currently, the communication between BMAL1 and HIF-1α is mostly explored using cell models, investigating the relationships between pathways or the regulatory relationships, with insufficient exploration in animal models. Therefore, we propose that the circadian rhythm disruption model (control of light-dark cycle),^28^ or circadian rhythm gene knockout^274^ combined with the bone defect model (mandibular defects,^275^ or tooth extraction^276^) can be used to explore the impact of circadian rhythm disorders on bone regeneration at the site of bone defect, and then study the interaction between BMAL1 and HIF-1α during the process of bone regeneration. In the previous part, we concluded that the current mainstream view holds that the crosstalk between BMAL1 and HIF-1α is manifested in three aspects: (1) BMAL1 and HIF-1α mutually regulate gene expression; (2) BMAL1 and HIF-1α bind to each other to form a heterodimer, regulating the expression of downstream genes; (3) There is overlap or mutual regulation between the downstream genes that each of BMAL1 and HIF-1α regulates, ultimately jointly regulating the physiological functions of the cells.

At present, there are still deficiencies in the research on each part. First, transcriptional changes in *Bmal1* and *Hif-1α* may not reflect direct regulation due to confounding factors. Under hypoxic conditions, the inhibition of ceramide kinase (a key enzyme in multiple cellular processes) leads to the autophagic degradation of BMAL1.^34^ HIF-1α can also enhance the amplitude of the circadian rhythm oscillation by directly binding to the promoter of Per2 (a gene involved in inhibiting the expression of BMAL1 in the circadian rhythm).^264^ Similarly, the changes in BMAL1 may cause changes in HIF-1α that are not directly regulated. BMAL1 can fine-tune the activity of HIF-1α through its role in mitochondrial metabolism and by regulating oxidative stress.^212^ The CRY (a gene involved in inhibiting the expression of BMAL1 in the circadian rhythm) protein negatively regulates HIF-1α by reducing its half-life and decreasing its binding to the promoter of target genes.^277,278^ It is recommended that in the research, methods such as dual luciferase should be used to exclude the interference of other factors and accurately verify the regulatory relationship between BMAL1 and HIF-1α.^195^

Secondly, the true role of the heterodimer formed by the binding of BMAL1 and HIF-1α proteins is still controversial. Under normal physiological conditions, BMAL1 binds to CLOCK,^25^ and HIF-1α binds to HIF-1β to exert its function.^15^ These dimer forms are the main forms in which the two exert their main functions. In the real hypoxic condition, how much and what proportion of the BMAL1-HIF-1α heterodimer is formed, and whether it has a regulatory effect on cellular physiology? According to a previous study, the binding of HIF-1α and HIF-1β formed a strong band, much higher than the binding of HIF-1α and BMAL1.^40^ Additionally, with the development of emerging technologies, there are more means to verify the protein-level interaction between BMAL1 and HIF-1α, such as using molecular interaction instruments to verify the binding situation, or using software to predict the binding sites of the two proteins, and then modifying/mutating the sites to observe the binding situation.

While we have endeavored to synthesize the current understanding of HIF-1α and BMAL1 crosstalk in bone regeneration, our review also reveals several critical knowledge gaps that warrant further investigation. A primary limitation pertains to the spatiotemporal dynamics of hypoxic and circadian regulation across different cell types. Specifically, the current literature lacks detailed mechanistic studies that delineate how the specific roles of HIF-1α and BMAL1 are fine-tuned within different cells, each of which experiences a distinct hypoxic intensity and duration due to their sequential recruitment into the regeneration process. While we have provided a conceptual framework, direct experimental evidence linking the dynamically changing oxygen gradient to cell-type-specific HIF-1α/BMAL1 activity and downstream functional outputs remains scarce. Furthermore, the precise molecular impact of differential hypoxic sensitivity among these cells on the functional interplay between the HIF-1α and BMAL1 pathways is still largely unexplored. Future research employing cell-type-specific genetic models, real-time oxygen biosensing, and time-series transcriptomic analyses across the phases of healing will be essential to move from a macroscopic model to a precise, mechanistic understanding of this complex regulatory axis.

After summarizing a large number of studies, we also found that the expression or function of BMAL1 is tissue-specific. The results obtained from the verification in one tissue may be different or even opposite in another tissue. Based on the experiments classifying alterations in cellular metabolism according to gene regulation across different tissues, we have summarized the findings in Table 2 to provide a reference for tissue-specific research.

**Table 2:** The tissue-specific metabolic alterations resulting from the regulation of Bmal1 in different tissues

Regulation	Tissue/Cells	Metabolic Regulation	Reference
Bmal1 Overexpression	Mouse adipose tissue	Increased creatine cycling; increased energy expenditure;	^294^
	Human astrocytes	Suppresses aerobic glycolysis and lactate production by the reduction in HK1 and LDHA protein levels	^227^
	Human BM-MSCs	Increase the activity of ALP, promote the expression of RUNX2, OSX, OPN, and OCN, and promote osteogenic differentiation	^295^
	MC3T3-E1	Enhanced calcium salt metabolism; increased mineralized nodules.	^296^
	RAW264.7	Enhance the expression of CTSK and NFATc1; conduct the glucocorticoid signal into the osteoclasts	^297^
Bmal1 Knockout	Mouse bone	osteoblast-specific Bmal1 KO mice: Increase the sensitivity of osteoblasts to 1,25(OH)₂D₃; decreased bone mass, cortical volume, trabecular number, and trabecular thickness in the tibia; decreased bone volume in the vertebrae	^171^
		osteoblast-specific Bmal1 KO mice: Higher levels of serum osteocalcin (which is known to regulate glucose homeostasis)	^170^
		osteoclast-specific Bmal1 KO mice: decreased expression of steroid receptor accessory activating factor family members (SRC-1, SRC-2, SRC-3); increased bone mass in the femur and vertebrae; reduced bone resorption capacity	^173^
	Mouse skeletal muscle	Glucose uptake is impaired, and the activities of muscle glucose transporter GLUT4 and key rate-limiting enzymes of glycolysis decrease; the conversion of citrate to isocitrate occurs in the TCA cycle; amino acids and genes regulating fat metabolism significantly increase	^298^
		Decreased glycolytic activity; increase in glucose-alanine cycling	^215^
	Mouse intestine	Transformation of lipid biosynthesis into gluconeogenesis at night; glucose production increases (hyperglycemia); insulin insensitivity	^299^
	Human embryonic stem cell-derived cardiomyocytes	Significantly attenuated mitochondrial oxidative phosphorylation and compromised cardiomyocyte function	^211^
	Mouse adipose tissue	Reduced expression of genes involved in creatine metabolism, decreased abundance of creatine	^294^
	Mouse liver	Enhanced lipolysis; reduced lipogenesis; diminished lipid uptake; decreased β-oxidation function of liver mitochondria	^300^
	Mouse renal tubule	Impairments in NAD+ biosynthesis, fatty acid transport, carnitine shuttle, and β-oxidation	^301^

## Conclusion

In this review, we summarize the key role of the inflammatory phase in bone regeneration and the significant roles of HIF-1α and BMAL1 in bone regeneration. During the inflammatory phase of bone regeneration, cells are in a hypoxic microenvironment, and how to carry out orderly life activities under such conditions is a challenge. On the one hand, the hypoxia response mediated by HIF-1α helps cells resist it, and on the other hand, BMAL1 promotes the resolution of inflammation and the progress of osteogenesis. Beyond their individual roles in hypoxia response and circadian rhythm, we emphasize their synergistic interaction. HIF-1α and BMAL1 exhibit close connections at multiple levels: genetic, protein, and downstream regulatory pathways. These connections collectively regulate intracellular expression changes and metabolic reprogramming to balance oxidative stress, glycolytic flux, and osteo-angiogenic coupling, ultimately ensuring the normal physiological activities of cells. Crucially, this interaction extends beyond physiological bone healing to pathological contexts. In osteoporosis (shift work- or diabetes-associated), osteoarthritis, and titanium implant osseointegration. Despite significant research progress, key challenges and limitations persist. In our view, the communication and interaction between HIF-1α and BMAL1 require more precise exploration. Future research should further investigate the spatiotemporal interplay between hypoxia response and circadian rhythm to unravel the underlying mechanisms of bone regeneration.
